# Collagen-Based Mechanical Anisotropy of the Tectorial Membrane: Implications for Inter-Row Coupling of Outer Hair Cell Bundles

**DOI:** 10.1371/journal.pone.0004877

**Published:** 2009-03-18

**Authors:** Núria Gavara, Richard S. Chadwick

**Affiliations:** Auditory Mechanics Section, National Institute on Deafness and Other Communication Disorders, National Institutes of Health, Bethesda, Maryland, United States of America; The University of Western Ontario, Canada

## Abstract

**Background:**

The tectorial membrane (TM) in the mammalian cochlea displays anisotropy, where mechanical or structural properties differ along varying directions. The anisotropy arises from the presence of collagen fibrils organized in fibers of ∼1 µm diameter that run radially across the TM. Mechanical coupling between the TM and the sensory epithelia is required for normal hearing. However, the lack of a suitable technique to measure mechanical anisotropy at the microscale level has hindered understanding of the TM's precise role.

**Methodology/Principal Findings:**

Here we report values of the three elastic moduli that characterize the anisotropic mechanical properties of the TM. Our novel technique combined Atomic Force Microscopy (AFM), modeling, and optical tracking of microspheres to determine the elastic moduli. We found that the TM's large mechanical anisotropy results in a marked transmission of deformations along the direction that maximizes sensory cell excitation, whereas in the perpendicular direction the transmission is greatly reduced.

**Conclusions/Significance:**

Computational results, based on our values of elastic moduli, suggest that the TM facilitates the directional cooperativity of sensory cells in the cochlea, and that mechanical properties of the TM are tuned to guarantee that the magnitude of sound-induced tip-link stretching remains similar along the length of the cochlea. Furthermore, we anticipate our assay to be a starting point for other studies of biological tissues that require directional functionality.

## Introduction

Biological tissue often achieves its function through anisotropic elastic properties. The mammalian inner ear seems to rely on an anisotropic extracellular matrix, the tectorial membrane (TM), to guide sound-induced vibrations to specific sensory hair cells. To understand the role of the TM in hearing it is helpful to outline the basic elements of the hearing process [1]. The mammalian hearing epithelium, the organ of Corti, sits inside the snail-shaped cochlea on the basilar membrane (BM). The BM is graded in stiffness along the cochlea and vibrates in response to sound-induced movements of the cochlear fluids. As a result, the stereocilia bundles of outer hair cells (OHCs) are sheared against the TM, which is situated over the sensory epithelium and spans the entire length of the cochlea. The OHCs can change their length through a piezo-electric mechanism when stereocilia are deflected [2]. Stereocilia deflection in OHCs and inner hair cells (IHC) stretches tip-links that open transduction channels, thereby inducing a receptor potential and modulating neurotransmitter release onto the postsynaptic spiral ganglion neurons [1].

Recent studies using mutant mice with altered TM organization have shown that its morphological anisotropy has a crucial role in mammalian hearing [3], [4], [5]. This acellular matrix contains two main groups of components, collagen fibrils and non-collagenous proteins. The latter compose a striated-sheet matrix surrounding the collagen fibrils [5]. Collagen fibrils are organized in thick fibers of ∼1 µm diameter that run nearly radially across the TM [6]. Surprisingly, the existence of TM's anisotropy in mammals is accompanied with a unique pattern and orientation of sensory cells, typically with one row of IHCs and three rows of OHCs [1]. Furthermore, the collagen fibers and the OHC stereocilia bundles display a coincident slanting with respect to the radial direction [7]–[9]. This suggests that the direction of collagen fibers in the TM coincides with the direction of stereocilia bundle deflection that leads to maximal sensitivity. Nevertheless, despite all these striking directional cues, the relevance of TM's mechanical anisotropy in hearing has not been established. This requires measurement of the anisotropic mechanical properties of the TM and subsequent modeling of the TM's mechanical interaction with hair cell stereocilia. It is important to emphasize that elastic moduli (as well as Young's modulus for an isotropic material) cannot be directly measured. An elasticity model is required to relate measurements of force and displacements to elastic moduli.

A number of morphological studies have noted the anisotropy of the TM and speculated about its function [4], [5], [10]. In spite of that, many studies aimed at measuring the mechanical properties of the TM have disregarded the prominent presence of the oriented collagen fibers, which renders the TM mechanically anisotropic [11]–[14]. Accordingly, the TM was modeled as an isotropic homogeneous material, and only one elastic modulus was reported. One recent study used Atomic Force Microscopy (AFM) to estimate the effective Young's modulus of the TM along different orthogonal directions [15]. That study did not provide knowledge of the actual anisotropic elastic moduli of the TM, since an anisotropic elasticity model was not used.

**Figure 1:**
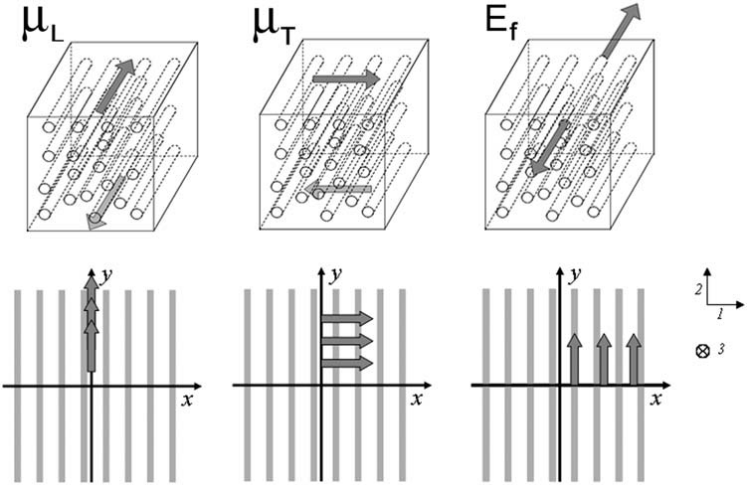
Elastic moduli of transversely isotropic fiber model. Upper row: the longitudinal shear modulus μ_L_ characterizes the elastic resistance to shear stresses applied to faces in a direction parallel to the fibers; the modulus μ_T_ characterizes the elastic resistance to shear stresses applied to faces in a direction perpendicular to the fibers; the fiber modulus E_f_ characterizes the resistance to tensile stresses applied to the fibers. In all cases, the larger the modulus the larger the resistance to deformation. Lower row left panel: the AFM probe exerts a shear force to the surface of the TM in the fiber direction and the resulting y-displacement of a fluorescent microsphere positioned at the center (not shown) is recorded, The measurement is repeated for different AFM tip-microsphere y-distances and the G_22_(0,y,0) formula (cf. Modeling) is fit by regression to extract μ_L_. Lower row middle panel: the AFM probe exerts a shear force to the surface of the TM perpendicular to the fiber direction and the resulting x-displacement of a fluorescent microsphere is recorded, The measurement is repeated for different AFM tip-microsphere y-distances and the G_11_(0,y,0) formula is fit by regression to extract μ_T_. Lower row right panel: the AFM probe exerts a shear force to the surface of the TM in the fiber direction and the resulting x-displacement of a fluorescent microsphere is recorded, The measurement is repeated for different AFM tip-microsphere x-distances and the G_22_(x,0,0) formula is fit by regression to extract E_f_ given μ_L_.

We have previously described the TM using the simplest model of anisotropy, the transversely isotropic model, in which there is a single family of parallel fibers embedded in a matrix whose elastic properties are the same in any direction perpendicular to the fibers [16] (Fig. 1). In such a model, the fibers correspond to the collagen fibers and the matrix corresponds to the striated-sheet matrix of non-collagenous proteins. When the material is incompressible, mechanical properties can be described by three elastic moduli, a fiber modulus (E_f_) and two shear moduli, one parallel to the fibers (μ_L_) and another perpendicular or transverse to them (μ_T_) [17]. The influence of these moduli are depicted in the upper row of Fig. 1, The larger the moduli the more difficult it is to deform the material in response to the applied stresses indicated by the arrows. Such elastic constants determine stress-strain relations, wave propagation speed, and the amount of stereocilia deflection when they are sheared against the TM.

Here we report a new technique to measure the anisotropic mechanical properties of the TM by combining AFM, fluorescence microscopy and modeling. The main technical contribution of the present work was unraveling which measurements to make on the surface of the TM to find the 3 moduli (see Fig. 1 lower row), and overcoming the essential difficulty in applying a calibrated shear force by the AFM cantilever in the surface plane of the TM. This novel approach has enabled us to measure for the first time the three anisotropic elastic moduli of the TM.

## Results

### A new technique to measure tissue anisotropy

**Figure 2:**
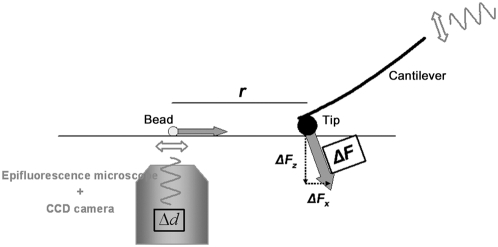
Setup to measure mechanical anisotropy. Point-like forces (*ΔF*) were imposed to indent the surface of the TM using a spherical tip. Since our experimental approach required controlled lateral forces, the AFM was tilted with respect to the stage. Surface displacements (*Δd*) were detected by tracking fluorescent beads deposited onto the surface of the TM. A set of measurements consisted of several indentation measurements performed at increasing tip-bead distances (*r*).

**Figure 3:**
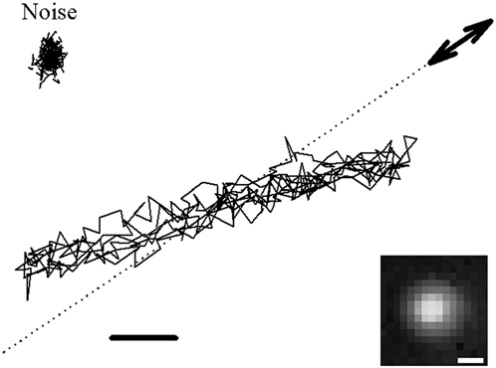
Representative bead position tracked in the plane. The bead moved back and forth as a result of an oscillating force applied on the TM. Double-headed arrow indicates the approximate direction of the applied force in the surface plane. A component of force was imposed in the z direction (perpendicular to the figure's plane). Dotted line shows the direction of the collagen fiber below the bead. Scale bar = 20 nm. The trajectory of the bead when no force was applied to the TM represents the measurement noise (upper left). Inset: Fluorescence image of the bead on the TM. Scale bar = 1 µm.

**Figure 4:**
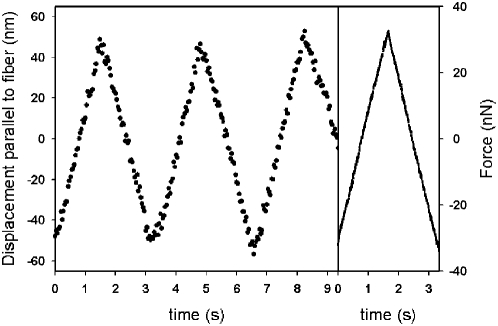
Time course of force imposition and bead displacement. *Right*. A representative oscillatory force applied to the TM using a 10 µm spherical tip. Offset force has been subtracted. *Left*. Resulting displacement of a bead attached to the TM in the direction parallel to the fiber. The bead was 8 µm away from the tip. Bead position was observed by fluorescence imaging and tracked with nanometer resolution using a Gaussian mask algorithm. Amplitude of force and displacement oscillation, as well as bead-tip distance, were then used for elastic moduli computation.

Elastic moduli determine the way a material deforms in response to an applied force. Analogous to the way a deformation pattern is transmitted along the surface of a bed when someone sits on it, deformations of the surface of the TM will also be observed when an AFM tip is used to impose forces on it. Clearly, those displacements must decay with increasing distance to the point of force application. A representative relation between applied forces, ΔF, and resulting displacements Δd, is 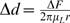, involving an elastic modulus and the distance r between the points of force application and observation. This kind of behavior for the transmission of deformations is found for a transversely anisotropic material. For such a material, the relationship between forces and deformations is described by a surface Green's tensor 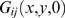, which contains the three elastic moduli of the material, and whose elements represent surface displacements in the *i* ^th^ direction that result from a unit point force at the origin acting in the *j* ^th^ direction. Interestingly, for a transversely isotropic material, G_ij_ = G_ji_,, i.e. Green's surface tensor is symmetric, so there are only six independent elements, albeit they are very mathematically complex in general. Along special directions however, some elements are given by simple functions that involve only one elastic modulus. We have used those tensor elements to readily estimate the elastic moduli of the material. Therefore, when designing our experimental setup (Fig. 2), the direction of forces that were applied as well as the direction of displacements measured, on the surface were carefully chosen to correspond to the tensor elements that provided direct computation of the elastic moduli (Fig. 1, lower row). It should be noted that the experimental design required forces to be exerted in the surface plane of the TM. Current AFMs, however, provide actual estimates of force only in the vertical direction. Therefore, we modified our AFM by placing it over a custom-made wedge, with a 10 degree tilt. By tilting the AFM head, we could apply a controlled component of the force in the plane of the stage (X–Y plane). Nevertheless, when the AFM head was tilted the applied force still retained a component in the vertical direction. Consequently, measurements were repeated in a non-tilted configuration to subtract the displacement resulting from vertical forces. The wedge could also be rotated, enabling the force on the X–Y plane to be directed parallel or perpendicular to the TM's fibers. To detect force-induced displacements, fluorescent beads were deposited onto the surface of the TM and tracked with nanometer resolution (Fig. 3 and 4). We then used our anisotropy model to compute the three elastic moduli from the applied forces, the observed bead displacements and the tip-bead distances.

### The TM exhibits marked anisotropy with larger elastic moduli at the base than at the apex

**Figure 5:**
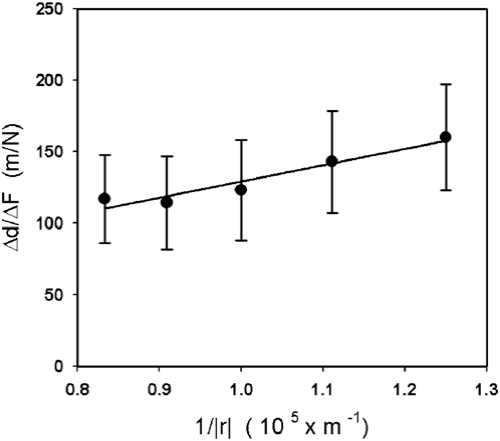
Algebraic decrease of the ratio between displacements and forces as the tip was moved away from the bead. Behavior of *Δd/ΔF* with increasing bead-tip distances (*1/|r|*). Solid line is the best linear fit, the slope of which yields an elastic modulus (*see* *Table 1*). This example graph shows the fit that gives the shear modulus perpendicular to the fiber in the apex of the cochlea. Samples from the same animal and cochlea location were averaged together for a single data point. Data are shown as mean±SE (N being the number of animals used).

For each measurement, the initial indentation was 1 µm and the cantilever was ramped with a sawtooth time pattern an additional micron in the same direction as the initial indentation (Fig. 4 right). Ramping indentations resulted in oscillatory forces with amplitudes ranging 10–120 nN. Consistently, the position of the fluorescent beads also displayed oscillatory movements with amplitudes ranging 20–200 nm (Fig. 3 and 4) at an angle with respect to the direction of the applied force. It should be noted that this angle arose from two different reasons. On the one hand, for certain configurations the Green's tensor predicts a component of displacement perpendicular to the force. On the other hand, small misalignments between the direction of force application and the direction of the fibers also contribute to this perpendicular component (Fig. 3). This angle did not enter into the calculation of the elastic moduli since only the component of displacement in the direction of the applied in-plane force component was required (*see Modeling section below*). As the tip was moved away from the bead during a set of measurements, forces did not change markedly, but resulting bead displacements were found to decrease. Therefore, 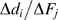 was observed to decrease with increasing tip-bead distances. In addition, decreases in 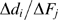 were well-fitted by a linear function of 1/|*r*|, indicating strong agreement between our model and the experimental data (*see Modeling in the Methods section* and Fig. 5). On additional measurements, we verified that the slope of the fit did not depend on the initial indentation (Fig. S1). The slopes of the fits were then used to compute the three elastic moduli. The fiber modulus was found to be on the order of a KPa, whereas the shear moduli were an order of magnitude smaller (Table 1). All elastic moduli displayed larger values at the base of the TM than at the apex.

**Table 1:** Elastic moduli at the base and apex.

	Apex	Base
**μ_L_** (Pa)	181±66	334±450
**μ_T_** (Pa)	72±8	205±170
**E_f_** (KPa)	1.82±0.72	5.39±11.13

### Estimation of other mechanical properties of the TM and relevance of collagen fiber slanting

Previous studies have reported various quantities that reflect certain extended mechanical properties of the TM. Such quantities include the propagation velocity of shear waves traveling longitudinally along the TM and the effective Young's modulus for an isotropic material. Using our modeling capabilities, we have derived formulas that relate shear wave speed (*C*) and effective Young's modulus (*E*) to the three elastic moduli that characterize a transversely isotropic material (Supplementary information S2):
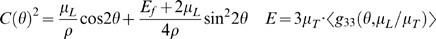
where ρ is the material's density and θ the slanting of the collagen fibers with respect to the radial direction. These formulas constitute in themselves theoretical results for this article. Table 2 shows the derived formulas, the estimates of *C* and *E* computed using the elastic moduli we have measured, as well as values previously reported in the literature. With one exception (reference [13]), our estimates of *E* lie in the range of the prior findings and reproduce qualitatively the gradient reported in the mechanical properties from base to apex in the TM. Comparison of the employed measurement methods and the results obtained can be found in the Discussion section. It should be noted that our estimates for shear wave speed represent a lower bound for this parameter, since viscoelasticity effects, which increase effective wave speeds [18], are not taken into account in our model. Accordingly, reference [18] observes similar apex-base gradients in shear wave speed, but reports greater values. Finally, our derived formula indicates that shear wave speed depends on the slanting angle of the collagen fibers. We compute that if collagen fibers were not slanted, longitudinally traveling waves would be observed to propagate ∼2-fold slower, according to the formula given in Table 2, which is derived in Supplementary information S2. Here we confer, a relevance to the long observed slanting of the collagen fibers.

**Table 2:** Other mechanical properties of the TM.

	Apex^a^		Base^a^	
	this work	others^b^	this work	others^b^
**Young's Modulus (KPa)**	0.78	0.53 [14]	1.93	1.9 [14]
		∼4.5 [12]		∼4.5 [12]
		∼25 [13]		∼215 [13]
**Wave speed (m/s)**	0.66	∼4 [18]	0.82	∼7 [18]

a *θ* values were 15° in the base and 25° in the apex [7].

b Numbers in brackets identify referenced paper.

### Larger elastic moduli at the base correlate with thicker collagen fibers and smaller gaps between them

**Figure 6:**
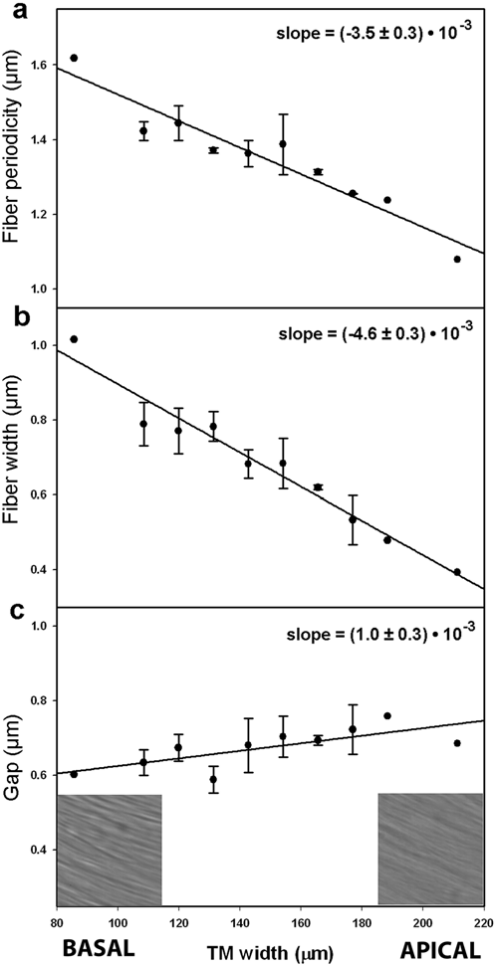
Changes in the morphology of collagen fibers in the TM from base to apex. Graphs show periodicity (*a*), thickness of fibers (*b*) and gap between fibers (*c*) with respect to TM's width. For each imaged sample, the width of the TM was measured and used as an estimate of the distance from the base of the cochlea. Narrower TM's were considered to be samples extracted from regions closer to the base. Samples from the same animal and cochlea location were averaged together for a single data point. Data are shown as mean±SE (N being the number of animals used). Insets: examples of DIC images of the surface of the TM facing the OHC recorded at the base and the apex. Size of images in insets is 21 µm.

Changes in the mechanical properties of the TM along the length of the cochlea were related to changes in the width or distribution of the collagen fibers composing the TM. The surface of the TM facing the sensory epithelium was imaged by DIC microscopy and the acquired images were then analyzed using an enhanced autocorrelation technique (Supplementary information S1, Fig. S3 and Fig. S4). We simultaneously measured fiber width and fiber periodicity, i.e. the distance between the centers of two neighboring fibers. In agreement with our finding that E_f_ was largest in the base, collagen fibers were found to be thickest in the basal region (1 µm). Fiber thickness then displayed a uniform decrease towards the apex (0.4 µm) (Fig. 6a). Fiber periodicity was also found to decrease from base to apex, but the slope of the decrease was smaller than that of fiber thickness (Fig. 6b). Consequently, fiber 1D density (fiber width/periodicity) also decreased from base to apex (data not shown). Finally, the gap between fibers was largest at the apex of the cochlea (0.75 µm at the apex vs 0.6 µm at the base) (Fig. 6c). Because this gap between collagen fibers is composed of striated-sheet matrix, larger gaps sizes at the apex agree with the smaller shear moduli we found at that location of the cochlea.

## Discussion

In this study we have used a novel technique to measure the anisotropic mechanical properties of the tectorial membrane. The elastic moduli are larger at the base of the cochlea, where collagen fibers were found to be thicker and the gap between them was smaller. The measured fiber modulus is an order of magnitude larger than the two shear moduli, resulting in a strong transmission of TM's deformations along the direction of the collagen fibers.

### Limitations of the method

We measured anisotropy of TM samples that were isolated from the tissues to which they normally attach and were then attached to a glass slide. Therefore, the sample preparation must be discussed to understand the applicability of our findings. Moreover, we will also briefly discuss the assumptions made for our model. The TM is very sensitive to the changes in the ionic concentration and the pH of its surrounding fluids [10], [19]. Such changes cause the TM to shrink or swell. TM's samples were submerged in artificial endolymph (AE) throughout dissection, storage and measurement. The AE composition we used was proposed by Shah *et al.* [19] and has become the standard bathing solution for studies on mechanical properties of the TM. Consequently, our results can be readily compared to other mechanical studies of the TM and to a great extent mimic the environment of the TM in *in vivo* conditions. It has been reported that the mechanical properties of the TM vary with time after dissection [11]. Although time variations can account for some of the variability of our results, they are too small to account for the observed difference in the mechanical properties between base and apex. The anisotropy model used is based on two assumptions, semi-infinite thickness and incompressibility of the material. The semi-infinite thickness assumption holds when the range of indentations used is much smaller than the thickness of the sample. The guinea pig's TM thickness in the studied region is in the range of 34 to 41 microns [20]. Using the lower value of this range, we estimate the amount of displacement, using our model, to decrease to 0.5% at the surface of the TM opposite to where the forces were imposed. Therefore, the attachment of the TM to the underlying glass had no effect, and the semi-infinite thickness assumption is justified (Fig. S2). Because the TM is a polyelectrolyte gel, the incompressibility assumption holds if the deformations are imposed fast enough to prevent changes in volume during one cycle. One indentation cycle took 3.3 seconds, while the time required for volume changes of the TM has been reported to be ∼1 hour [21]. On the other hand, the quasi-static approach used also guaranteed that no viscous forces contributed to our measurements. Finally, the TM's surface exhibits some curvature when laid flat onto a coverslip. According to our measurements, the tangent angle on the region studied was ∼5 degrees. Because the measurements were repeated in the tilted and non-tilted configuration and the resulting displacements were subtracted, the TM's angle does not affect the approach angle of the indenter. However, the remaining force component was directed on the plane of the coverslip, not the plane of the TM. This resulted in only a 0.4% (1-cos5°) overestimation of the force applied on the plane of the TM's surface.

### Comparison with previous techniques to measure mechanical properties of tissues

To estimate the anisotropic mechanical properties of a biological tissue, a suitable technique to apply controlled horizontal forces on the surface of the sample is required. Previous studies on the TM have pulled a magnetic bead to apply such forces along perpendicular directions [11]. Nevertheless, the uncertainty in the estimation of the contact area between the magnetic bead and the TM does not allow measurement of absolute stresses or elastic moduli. Similarly, a recent approach using lateral force application with an AFM tip, mixed cantilever twisting and slipping regimes, failing to obtain actual elastic moduli estimates [15]. Unlike common AFM or magnetic bead techniques to estimate stiffness, which measure sample deformation through cantilever bending or bead movement, our setup uses microscopic beads resting over a fiber to track surface deformation. By uncoupling the probe imposing force onto the surface from the probe tracking sample deformation we can measure the third elastic modulus, that is, the fiber modulus, for the first time. Estimation of this modulus would not be possible with a standard AFM alone or a rheometer. Finally, it should be noted that the estimates for radial and longitudinal stiffness or the elastic moduli along orthogonal directions previously reported [14], [15] are probe dependent or unrelated to an anisotropic solid model. These facts limit their applicability to the study of cochlear mechanics. By contrast, the three elastic moduli we report can be readily used in models of wave propagation in the cochlea to further our knowledge of the hearing process.

### Comparison to previous TM mechanical measurements

Young's modulus of the tectorial membrane has been measured by several groups in the past recent years, as is shown in Table 2. It should be noted that considerable effort was devoted to assess whether the TM displays graded stiffness along its length. In a previous paper, we could not statistically show differences on *E* from base to apex, although the measured values of *E* were in the range of those previously and subsequently reported [12]. On the contrary, Gueta *et al.* reported graded stiffness along the cochlea, but the observed magnitudes were at least an order of magnitude greater than all published data [13]. The discrepancy in the results may be explained by the small size of their probe, which has been shown to result in overestimations of *E* [22]. Richter *et al.* have been the only group so far to report Young's modulus of the TM *in situ* [14]. Interestingly, the effective Young's modulus we compute is in total agreement with their data, both in magnitude and gradient. It should be noted that their measurements reflect the combined stiffness of the spiral limbus (SL) and the TM, as it is felt by the OHCs' bundles. Since the two contributions are in series, the similarity between their and our results indicates that the Young's modulus of the SL attachment must be much larger than that of the TM. Similar mechanical properties for the SL have been indicated by others [18], [23]. Together, these results suggest that the mechanical properties relevant to the OHCs bundles are mainly those of the TM's body and not the SL.

### Relationship between probe size and stereocilia diameter

The main role of the TM in hearing is its mechanical interaction with the tips of the stereocilia of the OHC. The diameter of a single stereocilium or its initial indentation into the TM is in the hundreds of nanometers range [24]. By contrast, the amount of indentation and the effective contact area of the probe used in our experiments was on the micron range. To test the possible implications of this scale difference, we applied force ramps using different initial indentations and contact areas. Interestingly, we did not observe marked changes on the mechanical properties of the TM (Fig. S1). Therefore, our results reflect the anisotropic mechanical properties of the TM in the spatial scale relevant to hearing.

### TM's graded mechanical properties ensure that tip-link stretching remains similar along the length of the cochlea

We have previously shown that radial motion of the TM is responsible for sound-induced OHC stereocilia shearing and subsequent deflection [25]. According to our model and data, shear forces required to deform the TM in the radial direction scale as 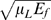 (*see modeling below*), yielding a 2.3-fold difference from base to apex. We can similarly estimate the amount of forces required to deflect stereocilia to produce equal amounts of tip-link stretching from base to apex. It should be noted that the upper end of the tip-link, the structure that mediates mechanotransduction, does not extend to the top of the stereocilium [1]. Therefore, tip-link stretching and resulting opening of ion-channels depends not on the lateral movement of the stereocilium tip but rather on its deflection angle. We have used the values of rotational stiffness reported by Kross [26] for the OHC1 and OHC2 stereocilia bundles on the second and fourth turn of the cochlea, which match the locations where our AFM measurements were performed. Because OHC bundles contain 3 rows of stereocilia all along the length of the cochlea [27], we can directly convert changes in bundle rotational stiffness to changes in single stereocilia rotational stiffness. The change in stereocilia rotational stiffness between the two selected turns is ∼1-fold for the OHC1 and ∼5-fold for the OHC2. Remarkably, the 2.3-fold difference we calculate is in the range of the rotational stiffness gradients reported experimentally. Therefore, we suggest that the mechanical properties of the TM are tuned to guarantee that despite the varying properties of the stereocilia bundle, sound-induced tip-link stretching remains similar along the length of the cochlea. Further investigation of this hypothesis will require anisotropy measurements to be performed at many more longitudinal and radial locations on the whole TM.

### TM's marked anisotropy facilitates the directional cooperativity of sensory cells, which could enhance the cochlear amplifier

**Figure 7:**
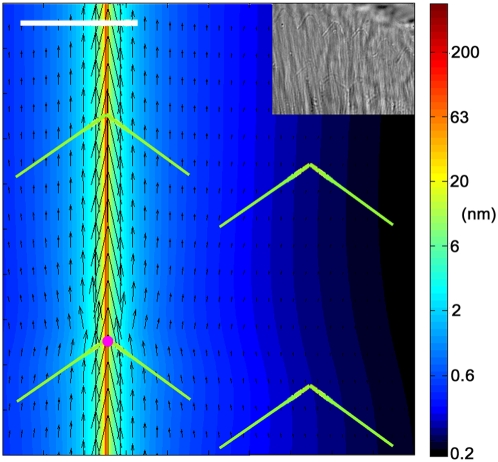
Surface deformation patterns of the TM. In this plot depicting the surface of the TM, the collagen fibers run vertically (not plotted) and the green inverted Vs represent the stereocilia bundles of 4 neighboring cells (only two of the three existing OHC rows are shown). A shear point force (20 pN) is applied to the surface of the TM, with the force oriented in the vertical direction (point of force application marked by the pink dot over the left bottom stereocilia bundle). As a result of the shear force, TM's deformation affects the neighboring stereocilia bundle located on a different row (vertical direction), but not the one located on the same row (horizontal direction). It should be noted that larger longitudinal coupling would be observed if the stimulus was a wave launched radially or semi-radially. The deformation field has been obtained using the elastic moduli measured at the base of the TM and the Green's tensor for a transversely isotropic model. Color scale indicates the magnitude (nm) of deformation. Arrows depict the direction and relative magnitude of deformation. Arrows are displayed with 600 nm spacing in the radial direction. Scale bar is 5 µm. Inset: DIC image of the surface of the TM facing the OHCs. The image shows the imprints of the stereocilia bundles of two rows of OHCs.

The mammalian TM and the arrangement of the sensory cells underneath it display striking directional similarities [7]–[9]. To expose how several key roles in mammalian hearing can be enhanced by the anisotropic mechanical properties of the TM we have combined the measured values of the elastic moduli and our modeling capabilities. Figure 7 shows the deformations of the TM's surface as a result of a point force imposed in the direction of the collagen fibers. We have used the position of the imprints obtained from our DIC images (Fig. 7, inset) to correctly orient the stereocilia bundles. Because of the TM's marked anisotropy, local deformations of the TM's surface decay very fast as we move perpendicular to the fibers (horizontal direction on the plot), but very slowly in the direction parallel to them (vertical direction on the plot). The strong radial coupling between neighboring cells of different rows ensures that the stereocilia of the three OHCs deflect in concert, so that their ion-channels are opened at the same time. This could radially synchronize the prestin-dependent changes in length of the OHCs, which are crucial for the amplification process required for proper hearing. On the other hand, it has been proposed that spontaneous oscillations of the stereocilia bundle can contribute to the cochlear amplifier. Accordingly, a model has been proposed to estimate the gains of different spatial arrangements of stereocilia bundles coupled together through an overlying soft membrane [28]. The large anisotropy of the TM that we report implies that the TM would act as a faithful transmitter of stereocilia bundle oscillations only to neighboring bundles on different rows. According to the model [28], this would boost the gain of a putative stereocilia bundle amplifier, resulting in a resonant system of triply-cooperative bundles. Type II spiral ganglion cells have been observed to innervate ∼60 cells, typically with different types of nerve terminals (afferent, efferent or reciprocal) for each cell row [29]. It has been hypothesized that such innervation system could give rise to a complex neural network in the auditory periphery [29]. Such apparent division of synaptic tasks at the OHC level could be assisted by the presence of the TM synchronizing the mechanotransduction events. Thus, the markedly anisotropic TM could perform multiple tasks of coordinating somatic and bundle contributions to cochlear mechanics, as well as influencing neural functions. Indeed, the TM serves as a suitable material to study mechanical anisotropy and its key role in biological tissue function. Furthermore, the sophisticated experimental technique that we have developed will significantly benefit the field of tissue mechanics by providing knowledge of local mechanical anisotropic properties.

## Materials and Methods

### Sample isolation

Tissue samples were acquired from 16 juvenile female pigmented guinea pigs (weight 150–200 g). Twelve animals were used for anisotropic mechanical measurements and four for morphology measurements. Several samples were used for each animal. All animals included in the study tested positive for pinna (startle) response. Tectorial membranes were isolated as previously outlined [12]. In brief, animals were rendered deeply unconscious by CO_2_ gas and then decapitated. Bullae were removed and transferred immediately to an artificial endolymph (AE) bath [19]. Under a dissection microscope, the cochlea was exposed and individual samples of the TM were isolated from the organ of Corti. Samples were then transferred and laid onto coverslips coated with Cell-Tak for subsequent measurement. Upon visual inspection, we discarded samples that appeared to be folded or damaged in any way. For anisotropy measurements, we only used samples attached with the cover net facing down. TM samples were classified as basal or apical based on the cochlear turn from which they were dissected. As a finer classification measure for the TM's morphology study, we also measured the distance from the edge of the marginal band to the ridge associated with the attachment of the TM to the spiral limbus [18]. Measurements were performed within 24 hours after tissue dissection. Samples remained submerged in the AE bath throughout this period. No marked differences were observed on the mechanical properties of the tissue samples during this 24 hours period. All animal procedures were conducted according to approved National Institutes of Health (NIH) animal protocol number 1186-07.

### Anisotropy measurements setup

Measurements were performed on the stage of a Bioscope II AFM (Veeco Metrology Inc., Santa Barbara, CA), coupled to an inverted fluorescence microscope (Fig. 2). The AFM was used to exert controlled forces applied to the surface of the TM. A latex bead (10 µm diam.) glued to a tipless V-shaped Au-coated silicon nitride cantilever (nominal spring constant 0.58 N/m) was used to indent the TM. The spring constant of the cantilevers was calibrated using the thermal fluctuations method [30]. The vertical displacement of the tip was controlled by the Z-piezo located in the AFM head. Forces applied to the TM were measured by the AFM's photodiode. Lateral displacements of the sample with respect to the tip were generated by the X–Y piezos located on the stage. At the beginning of the measurements, the angle between the fibers of the TM and the X–Y piezos of the stage was established. Knowledge of this angle enabled us to perform all sample displacements and forces impositions on the orthogonal coordinate system defined by the vector parallel to the fibers, the vector perpendicular to the fibers in the plane of the surface, and the vector normal to the surface. To enable the AFM to exert a controlled lateral force component, a custom-made PVC wedge was positioned between the stage and the AFM. The resulting AFM tilting was 10 degrees. The wedge-AFM ensemble could also be rotated, enabling the lateral force to be directed parallel or perpendicular to the TM fibers. Since the AFM contained the cantilever, the laser source and the photodiode, no relative displacement arose between these three components. Fluorescent carboxylated polystyrene beads (1 µm diam.) deposited onto the surface of the TM were used as markers to track TM deformations.

### Protocol

Before measurements, the relationship between photodiode signal and cantilever deflection was calibrated by taking a force-displacement curve at a bare region of the glass coverslip and measuring its slope. To start the experiment, a fluorescent bead located in close proximity to the Hensen's stripe on the region of the TM located over the OHCs was chosen. Then, a phase contrast image was acquired to compute the angle of alignment of the fibers below the bead. During the experimental protocol five subsets of measurements were performed. On the first 2 measurement subsets the AFM was not tilted, and the sample was moved away from the tip in directions parallel (away from Hensen's stripe) and perpendicular to the fibers. Subsequently, the AFM was tilted so that the lateral force component was exerted in parallel to the fibers. Again, the sample was laterally moved away in parallel and perpendicular directions. Finally, the AFM was tilted to exert the lateral force component perpendicular to the fibers in the plane defined by the TM surface, and the sample was moved away only parallel to the fiber. A measurement subset consisted of 5 indentation measurements performed at increasing tip-bead distances, starting at 8 µm using 1 µm increases. Each time the sample was moved, the contact point between the tip and the sample was assessed by ramping the cantilever in the vertical direction at a constant speed (3 µm amplitude, 0.3 Hz, 1 µm indentation). The contact point was visually estimated using the force-displacement curves. Indentation measurements were performed at 1 µm initial indentation, with a 1 µm amplitude triangular ramping wave at 0.3 Hz frequency. A force-displacement curve was recorded for one cycle at 512 points per cycle, whereas images of the fluorescent bead were recorded at 25 frames/sec using a 40× objective and a CCD camera for a minimum of 3 ramping cycles.

### Data processing

Applied force was calculated from AFM recordings, using the cantilever bending and its spring constant. Amplitude of force changes (*ΔF*) were measured by using the approach direction of the force-displacement curve and computing its slope. The product of the slope and the length of one half cycle corresponded to *ΔF*. Sample deformation was measured by identifying changes in the position of the center of mass of the fluorescent bead from the previously stored images. The center of mass of the bead was determined by a Gaussian mask algorithm [31]. After correcting for slow drifts of the system, the position of the bead over time resembled the triangular pattern of the applied force. The triangular wave was then divided in cycles, and bead positions corresponding to the same temporal instant on different cycles were averaged together. The slope of the resulting curve times the length of one half cycle was used as amplitude of bead displacement (*Δd*). To test the tracking algorithm, experiments were performed using fluorescent beads attached to a slide and moved by a piezo system. Results of the experiments indicate that the accuracy of *Δd* computation is 1.3 nm, corresponding to 3.5% variability. All the computations were performed using Matlab (The Mathworks). In order to assert sample fiber deformation due solely to lateral forces, displacements measured on the non-tilting configuration were subtracted from displacements measured on the tilting configuration. The ratio of lateral forces (*ΔF_L_*) to resulting lateral displacements (*Δd_L_*) was computed as follows:
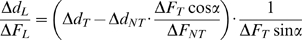
where α is the angle by which the AFM was tilted (10°), and the T and NT subscripts indicate tilting and non-tilting configuration, accordingly.

### Modeling

The Green's tensor 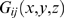 resulting from the Boussinesq-like solution for an anisotropic material provides 6 independent relationships between point forces, elastic moduli and resulting displacements along each axis [16]. In our experiments, the direction of applied forces and the selected component of the resulting displacements were conveniently paired in order to reproduce the elements of the Green's tensor that provided direct estimates of the elastic moduli (Fig. 1, lower row). The following relations were used to compute μ_L_, μ_T_ and E_f_:
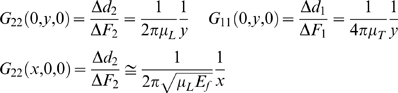
where 1 and 2 or x and y denote perpendicular to the fiber and parallel to the fiber in the surface plane, respectively. The third formula was also used to compute the stereocilium-induced TM's deformation discussed in connection with tip-link stretching.

## Supporting Information

Figure S1 Effect of initial indentation on elastic moduli computation. a: Force ramps were applied at the same locations of the TM using different initial indentations but equal oscillation amplitudes (500 nm). Force was applied perpendicular to the surface of the TM. Initial indentations used were: 500 nm (black), 1000 nm (red), 1500 nm (green), 2000 nm (yellow) and 2500 nm (blue). Behavior of Δd/ΔF with increasing bead-tip distances (1/|r|) was fitted to a linear function for each family of data. Solid lines are the best linear fit to the data family with matching color. Dotted line corresponds to the linear fit of all the data points pooled together. b: Slopes of the linear fits shown above. Data plotted as mean±SD provided by the fit.
(2.73 MB TIF)

Figure S2 Decay of sample deformation through the depth of the TM. Relative behavior of Δd/ΔF with increasing depth. Δd/ΔF was set to one at the TM's surface. The decay was computed on a location 1 µm distant from the point of force application.
(1.93 MB TIF)

Figure S3 Image processing procedure to compute fiber thickness and periodicity. a: bandpass filtered line. b: normalized autocorrelation of the derivative line. Inset: detail of the first positive and negative peaks of the autocorrelation. Red line indicates fit with a second order polynomial to obtain the refined peak location.
(2.70 MB TIF)

Figure S4 Results of the simulations. a: measurement of fiber periodicity. b: measurement of fiber thickness.
(3.11 MB TIF)

Supporting Information S1 (0.03 MB DOC)

Supporting Information S2 (0.05 MB DOC)
