# Supplementary figures and images for: Collagen-Based Mechanical Anisotropy of the Tectorial Membrane: Implications for Inter-Row Coupling of Outer Hair Cell Bundles
Source: PLoS One. 2009 Mar 18;4(3):e4877. doi: 10.1371/journal.pone.0004877 (PMC2654110; doi:10.1371/journal.pone.0004877)

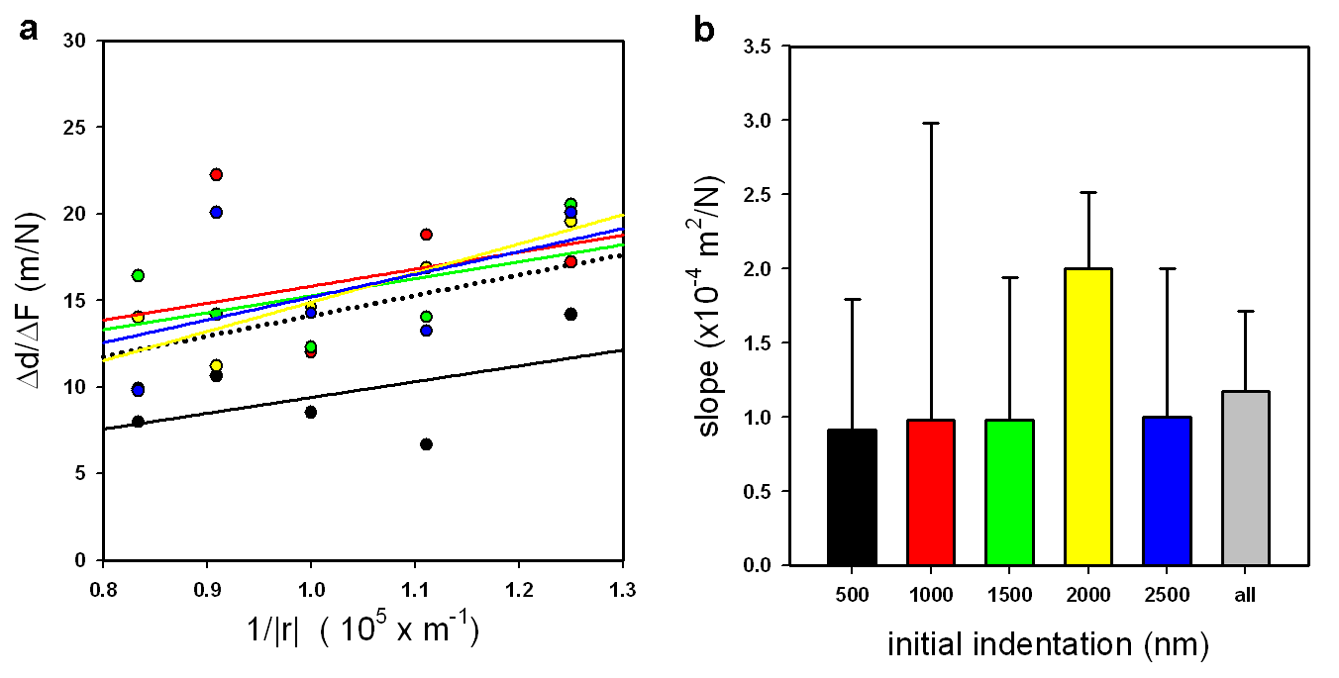

Supplement: Figure S1 — Effect of initial indentation on elastic moduli computation. a: Force ramps were applied at the same locations of the TM using different initial indentations but equal oscillation amplitudes (500 nm). Force was applied perpendicular to the surface of the TM. Initial indentations used were: 500 nm (black), 1000 nm (red), 1500 nm (green), 2000 nm (yellow) and 2500 nm (blue). Behavior of Δd/ΔF with increasing bead-tip distances (1/|r|) was fitted to a linear function for each family of data. Solid lines are the best linear fit to the data family with matching color. Dotted line corresponds to the linear fit of all the data points pooled together. b: Slopes of the linear fits shown above. Data plotted as mean±SD provided by the fit. (2.73 MB TIF) [file pone.0004877.s001.tif]

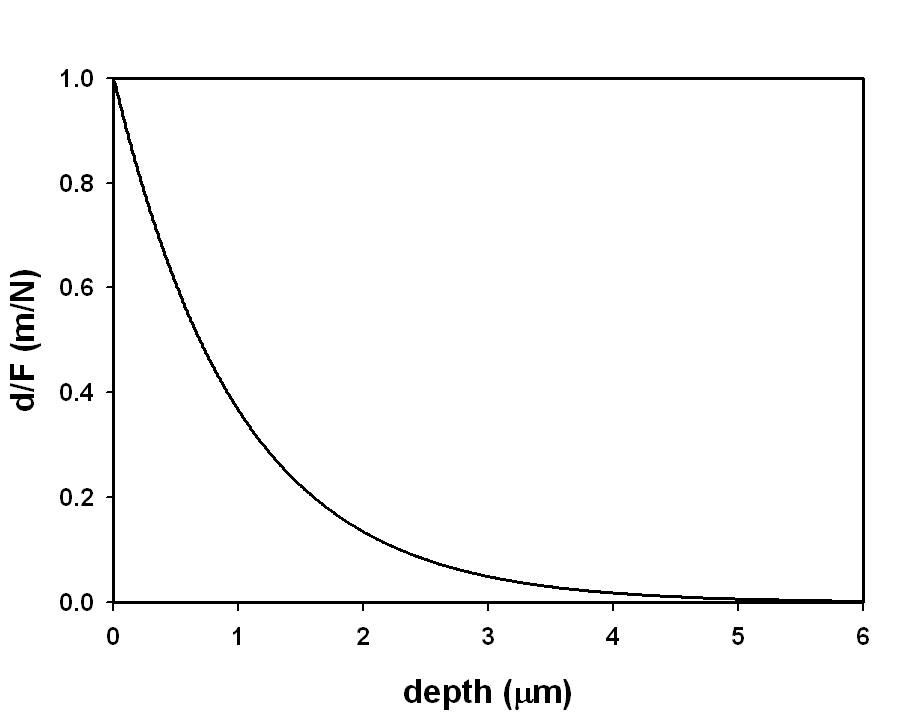

Supplement: Figure S2 — Decay of sample deformation through the depth of the TM. Relative behavior of Δd/ΔF with increasing depth. Δd/ΔF was set to one at the TM's surface. The decay was computed on a location 1 µm distant from the point of force application. (1.93 MB TIF) [file pone.0004877.s002.tif]

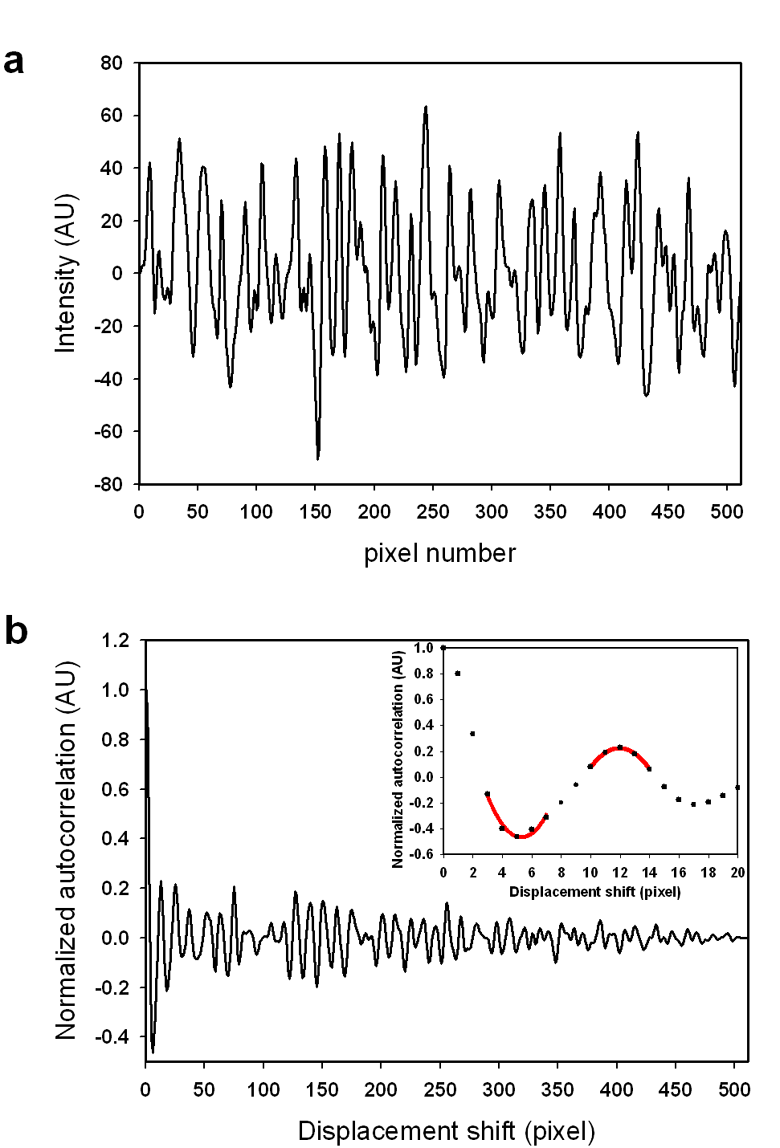

Supplement: Figure S3 — Image processing procedure to compute fiber thickness and periodicity. a: bandpass filtered line. b: normalized autocorrelation of the derivative line. Inset: detail of the first positive and negative peaks of the autocorrelation. Red line indicates fit with a second order polynomial to obtain the refined peak location. (2.70 MB TIF) [file pone.0004877.s003.tif]

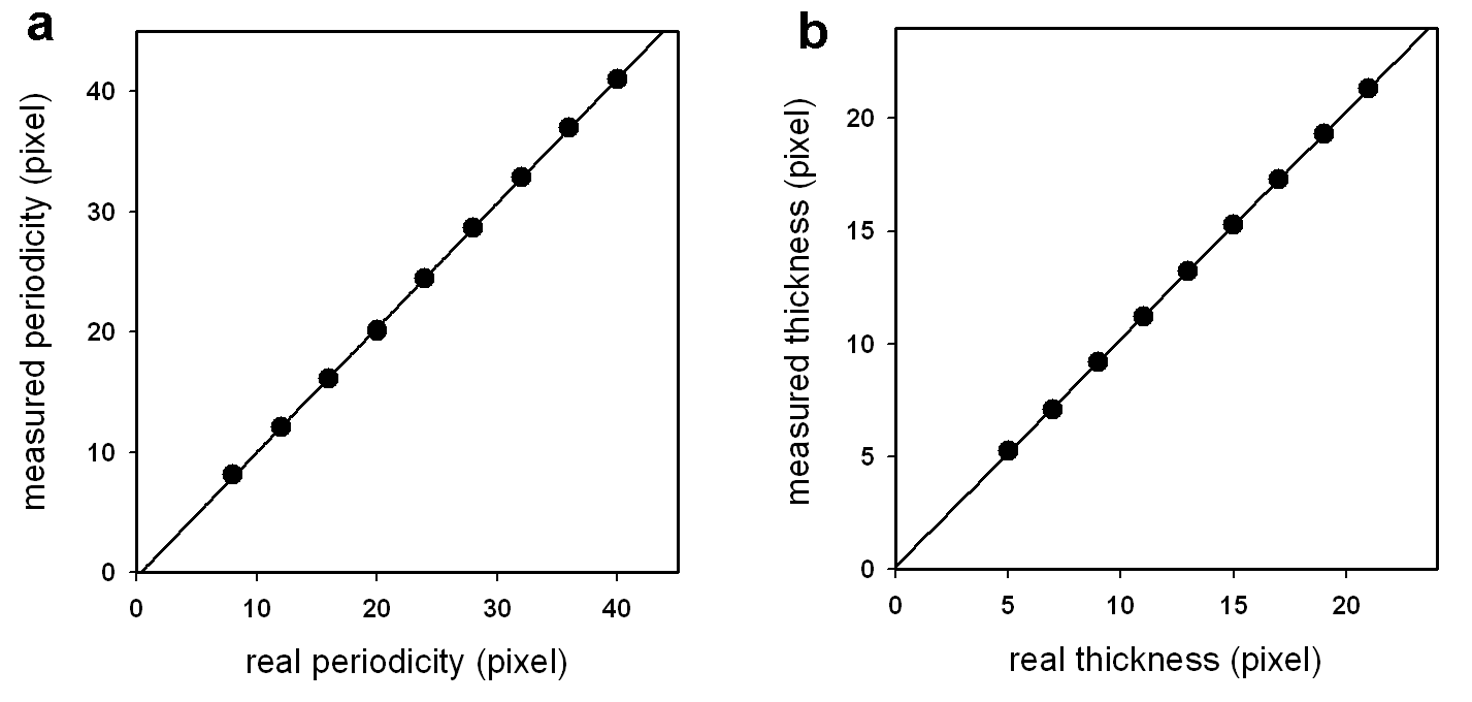

Supplement: Figure S4 — Results of the simulations. a: measurement of fiber periodicity. b: measurement of fiber thickness. (3.11 MB TIF) [file pone.0004877.s004.tif]
