# Supplementary material for: Collagen-Based Mechanical Anisotropy of the Tectorial Membrane: Implications for Inter-Row Coupling of Outer Hair Cell Bundles
Source: PLoS One. 2009 Mar 18;4(3):e4877. doi: 10.1371/journal.pone.0004877 (PMC2654110; doi:10.1371/journal.pone.0004877)
Supplement: Supporting Information S1 — (0.03 MB DOC) [file pone.0004877.s005.doc]

**Supporting information S1. Collagen fiber morphology measurements.**

*Collagen fiber imaging.* An initial image of the whole TM sample was acquired using a 10× objective to determine TM’s width. For each sample, the camera was manually rotated in order to image the fibers of the TM perpendicular to the vertical axis of the camera. Then, images of the surface of the TM were acquired using a 63× oil objective and high contrast DIC microscopy.

*Image processing.* For each stored image, vertical lines containing 512 pixels and corresponding to areas focusing the surface of the TM were extracted. Each line was bandpass filtered (Fig S2, *a*) to remove camera noise and non-uniform illumination and its numerical derivative was then performed. The derivative line was autocorrelated and the location of the first positive and negative peaks was established (Fig S2, *b*). Refined peak location was obtained by fitting the vicinity of the peak with a second order polynomial (Fig S2, *b inset*). The location of the first positive peak with respect to the central peak (zero displacement) corresponds to the periodicity of the fibers, whereas the location of the first negative peak with respect to the central peak corresponds to the width of the fibers. For each TM sample, a minimum of 500 lines were computed and the locations of their autocorrelation peaks were pooled together. Medians of the peaks distribution were used as an estimate of fiber periodicity and fiber width for each TM sample. Finally, the angle of the TM’s fibers with respect to the vertical axis of the image was computed to refine fiber positioning and obtain a real estimate of fiber morphological properties.

*Simulations*. We performed simulations to test our algorithm to compute fiber thickness and periodicity. Simulated lines contained 512 pixels and mimicked the oscillatory intensity patterns found in real lines. Periodicity and thickness of the fibers displayed in the lines could be adjusted. The peak-to-peak intensity of the oscillation had a Gaussian distribution (mean = 1 AU, SD = 1 AU) and additional white noise (SD=0.1 AU) was later added to each pixel. For each periodicity and thickness value, 500 lines were simulated and fed into the same algorithm used to process real images. The results of the simulations are displayed in figure S3 and show that our algorithm could faithfully measure variations in fiber thickness and periodicity.
