# Supplementary material for: Collagen-Based Mechanical Anisotropy of the Tectorial Membrane: Implications for Inter-Row Coupling of Outer Hair Cell Bundles
Source: PLoS One. 2009 Mar 18;4(3):e4877. doi: 10.1371/journal.pone.0004877 (PMC2654110; doi:10.1371/journal.pone.0004877)
Supplement: Supporting Information S2 — (0.05 MB DOC) [file pone.0004877.s006.doc]

**Supporting information S2. Derivation of other mechanical quantities.**

**1. Derivation of the effective Young’s modulus E_eff_ for a transversely isotropic material with a single family of fibers.**

$G_{33}(r,\theta+\frac{\pi}{2})=-\frac{1}{2\pi\mu_{T}r}\frac{\beta\cos^{2}\theta\sin^{2}\theta}{\beta\left|\cos\theta\right|\sin^{2}\theta\left(4\cos^{2}\theta+\beta\sin^{2}\theta\right)+4\left(\left|\cos\theta\right|-\sqrt{\cos^{2}\theta+\beta\sin^{2}\theta}\right)}+O\left(\sqrt{\frac{\mu_{T}}{E_{f}}}\right)$Consider the surface z-displacement w(x,y,0) produced by a point force F acting at the origin and normal to the surface defined by the plane (x,y,0) of a transversely isotropic half-space, with fibers aligned in the y-direction. This corresponds to the G_33_ component of the surface Green’s tensor defined in Chadwick et al. (2004). For the case of large fiber modulus E_f_ compared to the shear modulus *μ_T_*, the asymptotic form of G_33_ is

A2-1

For an isotropic material the result is simply

$G_{33}(r)=-\frac{1}{2\pi\mu_{eff}r}$ A2-2

Equating the mean values of G_33_ from A2-1 and A2-2 at a distance *r* yields

$\mu_{eff}=\frac{\mu_{T}}{\int_{0}^{2\pi} \frac{\beta\cos^{2}\theta\sin^{2}\theta d\theta}{\beta\left|\cos\theta\right|\sin^{2}\theta\left(4\cos^{2}\theta+\beta\sin^{2}\theta\right)+4\left(\left|\cos\theta\right|-\sqrt{\cos^{2}\theta+\beta\sin^{2}\theta}\right)}}$ A2-3

For an incompressible isotropic material the effective Young’s modulus E_eff_ = 3 μ_eff_.

**2. Derivation of shear wave speed formula for a transversely isotropic material with a single family of fibers.**

Consider displacements (u,v) and pressure p in the (x,y) plane, with fibers aligned in the y-direction. The dynamic equations of motion (Chadwick et al. 2004) are simplified for motion independent of z:

$\rho\frac{\partial^{2}u}{\partial t^{2}}=(2\mu_{T}-\mu_{L})\frac{\partial^{2}u}{\partial x^{2}}+2\mu_{L}\frac{\partial^{2}u}{\partial y^{2}}-\frac{\partial p}{\partial x}$ A1-1

$\rho\frac{\partial^{2}v}{\partial t^{2}}=2\mu_{L}\frac{\partial^{2}v}{\partial x^{2}}+(E_{f}+3\mu_{L}-2\mu_{T})\frac{\partial^{2}v}{\partial y^{2}}-\frac{\partial p}{\partial y}$ A1-2

$\frac{\partial u}{\partial x}+\frac{\partial v}{\partial y}=0$ A1-3

where ρ is the density and the three elastic moduli have been defined in the text. Substitution of

$\left[\begin{matrix} u \\ v \\ p \end{matrix}\right]=\left[\begin{matrix} U \\ V \\ P \end{matrix}\right]Exp[i(\omega t-k_{x}x-k_{y}y)$ A1-4

for a wave with wavenumber $\vec{k}:(k_{x},k_{y})$ yields the homogeneous linear algebraic system:

$\left[\begin{matrix} \omega^{2}-(2\mu_{T}-\mu_{L})k_{x}^{2}+\mu_{L}k_{y}^{2} & 0 & ik_{x} \\ 0 & \omega^{2}-\mu_{L}k_{x}^{2}+(E_{f}+3\mu_{L}-2\mu_{T}k_{y}^{2} & ik_{y} \\ k_{x} & k_{y} & 0 \end{matrix}\right]\cdot\left[\begin{matrix} U \\ V \\ P \end{matrix}\right]=\left[\begin{matrix} 0 \\ 0 \\ 0 \end{matrix}\right]$ A1-5

which has a solution only if the determinant of the matrix of coefficients is zero. Setting the determinant to zero yields the dispersion relation:

$\rho\omega^{2}(k_{x}^{2}+k_{y}^{2})=\mu_{L}(k_{x}^{4}+k_{y}^{4})+(E_{f}+2\mu_{L})k_{x}^{2}k_{y}^{2}$ A1-6

Introduce the propagation direction θ measured with respect to the x-direction:

$$\begin{matrix} k_{y}=k\sin\theta\\ k_{x}=k\cos\theta\end{matrix}$$

which yields the shear wavespeed *c(θ)*:

$c^{2}(\theta)=\frac{\omega^{2}}{k^{2}}=\frac{\mu_{L}}{\rho}\cos2\theta+\frac{E_{f}+2\mu_{L}}{4\rho}\sin^{2}2\theta$ A1-7
